# Effect of exercise therapy in patients with hip osteoarthritis: A systematic review and cumulative meta-analysis

**DOI:** 10.1016/j.ocarto.2023.100338

**Published:** 2023-01-19

**Authors:** Carolien H. Teirlinck, Arianne P. Verhagen, Leontien M. van Ravesteyn, Elja A.E. Reijneveld-van de Vendel, Jos Runhaar, Marienke van Middelkoop, Manuela L. Ferreira, Sita MA. Bierma-Zeinstra

**Affiliations:** a Dept. General Practice, Erasmus MC University Medical Center Rotterdam, the Netherlands; b Discipline of Physiotherapy, Graduate School of Health, University of Technology Sydney, Australia; c Sydney Musculoskeletal Health, The Kolling Institute, School of Health Sciences, Faculty of Medicine and Health, The University of Sydney, Australia

**Keywords:** Exercise therapy, Osteoarthritis of the hip, Cumulative meta-analysis, Clinical relevance

## Abstract

**Objective:**

To evaluate the existing evidence on the effect of exercise therapy in patients with hip osteoarthritis (OA) compared to no treatment and explore whether a further trial will change the current evidence.

**Design:**

Systematic review and cumulative meta-analysis using randomized controlled trials (RCT) to determine the effect on pain and function post-treatment, and at 6–9 months after treatment. Standardized mean difference (SMD) ​≤ ​−0.37 was considered clinically worthwhile. Extended funnel plots were used to simulate the impact of a new trial on the pooled effect size of pain and function.

**Results:**

18 RCTs were included. Post-treatment we found a beneficial effect of exercise therapy on pain (SMD -0.38, 95% Confidence Interval (CI): 0.55 to −0.22) and function (SMD -0.31, 95% CI -0.49 to −0.11). A beneficial effect of exercise therapy on pain (SMD -0.23, 95% CI: 0.41 to −0.05) and function (SMD -0.29, 95% CI: 0.45 to −0.12) was found 6–9 months after treatment. Most effect estimates were small, and it is unclear whether these are clinically meaningful. Extended funnel plots and a simulation of a new trial showed that only a new trial with a larger effect than the current pooled effect or a trial including 74,843 participants would change the pooled effect estimate from an unclear to a clearly clinically worthwhile effect.

**Conclusions:**

We found a beneficial effect of exercise therapy on pain and function in hip OA. It is unlikely a new trial added to current evidence will change the conclusion.

## 1 Introduction

Exercise therapy is recommended as a conservative treatment of osteoarthritis (OA) based on multiple meta-analyses and randomized trials [[1], [2], [3]]. Especially for knee osteoarthritis, the volume of evidence of its effectiveness is overwhelming [4]. A recent Cochrane review included 54 studies in their review of exercise therapy for knee OA [5]. Fewer trials have been conducted for the effect of exercise therapy on hip OA; a Cochrane review included 10 studies, published in 2014 [6]. Despite this lower number of trials, they found a statistically significant and clinically relevant reduction in pain and disability immediately after treatment and these beneficial effects were still present 3–6 months after treatment.

Ideally, we practice medicine based on the most current evidence available. Therefore, Cochrane reviews are updated every few years. In the case of an inconclusive review, new trials can be of great value. However, the beneficial effect of exercise therapy on pain and disability in patients with hip OA was well established in 2014 [6]. Will new trials be able to change the conclusion? For knee OA, we recently published a cumulative meta-analysis of the effect of exercise therapy [7]. We showed that no further trials conducted comparing exercise therapy to minimal or no treatment would likely change the current conclusions. Although fewer trials for this comparison in hip OA have been conducted, the Cochrane review already stated in 2014 that further research is unlikely to change the confidence in the estimate of effect but still new trials are conducted [6]. Evidently, we do not want to waste research resources or time spent on unnecessary trials. Moreover, it is unethical to randomize patients to an ineffective treatment. A cumulative meta-analysis and extended funnel plots provides more insight into whether further trials are needed and if so, what sample size should be recommended.

Therefore, our aim is to evaluate the existing evidence of the effect of exercise therapy compared to no or minimal treatment and to explore, by using a cumulative meta-analysis, if more research is needed.

## 2 Methods

Our department performed an update of the three Cochrane reviews [5,6,8] on the effect of exercise therapy in patients with hip and knee OA, commissioned by the National Health Care Institute of the Netherlands. This, to evaluate if exercise therapy for hip and knee OA patients should be covered by the basic health insurance in the Netherlands. For the current systematic review, we only used the studies on hip OA.

### 2.1 Main outcomes

Main outcomes were pain and function post-treatment and at 6–9 months after treatment.

### 2.2 Selection

A literature search was conducted in Cochrane Central Register of Controlled Trials (CENTRAL), MEDLINE, EMBASE, CINAHL, PEDro (Physiotherapy Evidence Database) and Web of Science from the date of last search in the Cochrane reviews until September 2021. We used the same search terms as the Cochrane reviews. No limits were set for language. References were searched for relevant studies. The full search can be found in the supplement.

We selected randomized controlled trials with the following characteristics: (P) adult patients (>18 years old) with clinical and/or radiological hip osteoarthritis, (I) the intervention was an active form of exercise therapy under supervision of a (physical) therapist, the intervention was not part of a multidisciplinary or multimodal program and was evaluated as a standalone intervention, (C) the intervention in the control group was usual care (like medication and/or education), no treatment or waiting list, and (O) outcomes were pain and/or function and were measured at short term (directly after end of treatment) and/or at long term (6–9 months after end of treatment). Studies evaluating interventions as hot packs, transcutaneous electrical nerve stimulation, ultrasound or likewise were excluded.

Selection of studies was done by two authors independently of each other (CHT, ERvdV or LMvR). First, titles and abstract were screened and selected for full-text reading. Secondly, the full texts were screened for inclusion. In case of disagreement, a consensus was reached, or disagreement was solved by a third author (APV).

### 2.3 Risk of bias assessment

Two authors assessed risk of bias independently (ERvdV, CHT, LMvR, MvM or APV). A third author (MvM or APV) assessed risk of bias in case of disagreement and no consensus. We used the same Cochrane risk of bias tools as was used in the original Cochrane reviews [5,6], in which on 7 domains a judgement of low, high or unclear risk was given. The 7 domains are random sequence generation, allocation concealment, blinding of participants and personnel, blinding of outcome assessment, incomplete outcome data, selective outcome reporting and other bias. In addition, studies were assigned an overall risk of bias. A study was considered to have a low risk of bias if the randomization procedure was done with a random sequence generation, proper allocation concealment, and intention-to-treat analysis was used. Studies were assigned a high risk of bias if less than 3 domains were assigned a low risk of bias. All other studies were assigned a moderate risk of bias [5,6,8].

### 2.4 Data extraction

Data extraction was done by two review authors (CHT, LMvR or APV) independently of each other using a standardized form. Disagreement was solved by consensus. The following data were collected: patient population (radiologic and/or clinical hip OA, OA severity), type of intervention (land-based, water-based, individual or group treatment, duration, and intensity), control group (usual care, education, no treatment), results (means and standard deviations) on pain and function post-treatment and at 6–9 months after the intervention. Standard errors or 95% confidence intervals (95% CI) were converted to standard deviations. If only change data were presented, these were extracted. If multiple instruments were used to measure pain or function, we used the instrument that was used by most studies in the analysis. If a trial included hip and knee OA patients and no data for hip OA patients separately were given, we contacted the first author to provide us the data for the analysis. Alternatively, data provided in the Cochrane Reviews were used.

### 2.5 Data analysis

Statistical pooling was done using Review Manager 5.3. We used a random-effect model assuming clinical heterogeneity. Outcomes were presented as standardized mean differences (SMD) with a 95% CI. We used a SMD of −0.37 as the threshold for a ‘clinically worthwhile effect” [9] where the negative value indicates outcomes in favor of exercise (less pain and better function). A funnel plot was created to observe possible publication bias. For the cumulative meta-analysis, we ordered the studies by publication date and added each study for a pooled result. Forest plots were created in SPSS version 24.

We used the GRADE approach to grade the quality (or certainty) of evidence of each outcome [10]. Quality of evidence was considered high if at least 2 studies were included. Subsequently, this could be lowered to moderate, low or very low quality of evidence if one or more of the following occurred: study limitations (>25% of patients are from studies with an overall high risk of bias), inconsistency of effect (statistical heterogeneity I^2^ ​> ​40% or ​< ​75% of patients show result in the same direction), indirectness (results are not suitable to extrapolate to the target population according to expert authors JR and SMAB-Z), imprecision (<400 patients in the analysis), other like publication bias or ‘fatal flaw’ (for example selective loss of follow-up) [[11], [12], [13]].

Stata version 15.1 was used to create extended funnel plots for the outcomes pain and function. In these funnel plots regions are calculated to evaluate the influence of a new study on the overall effect estimate, calculated by the meta-analysis. These regions indicate how large a new study and the effect estimate should be to significantly influence the overall effect estimate [14,15]. The red region means that the new study added to the current pooled effect would generate a new overall effect estimate and 95% CI that were clearly clinically worthwhile effect (new estimate and 95% CI ​≤ ​−0.37). The blue region indicates that a study added to the current pooled effect would generate a new overall effect estimate and 95% CI that were not a clinically worthwhile effect (new estimate and 95% CI ​> ​-0.37). The green region means a new study would yield a final pooled effect of uncertain clinical significance.

Finally, we simulated an extra cumulative meta-analysis, using the included trials and added a fictional new trial. The effect estimate of this new trial is equal to the current overall effect estimate of pain directly after treatment of the included trials. Step by step, we raised the number of participants, until the new overall effect estimates reached the clearly clinically worthwhile SMD (upper limit of the 95% CI ​≤ ​0.37). In this manner, we estimated the sample size of this fictional new trial that would ensure a clearly clinically worthwhile effect, given the current pooled effect.

## 3 Results

### 3.1 Selection

**Fig. 1:**
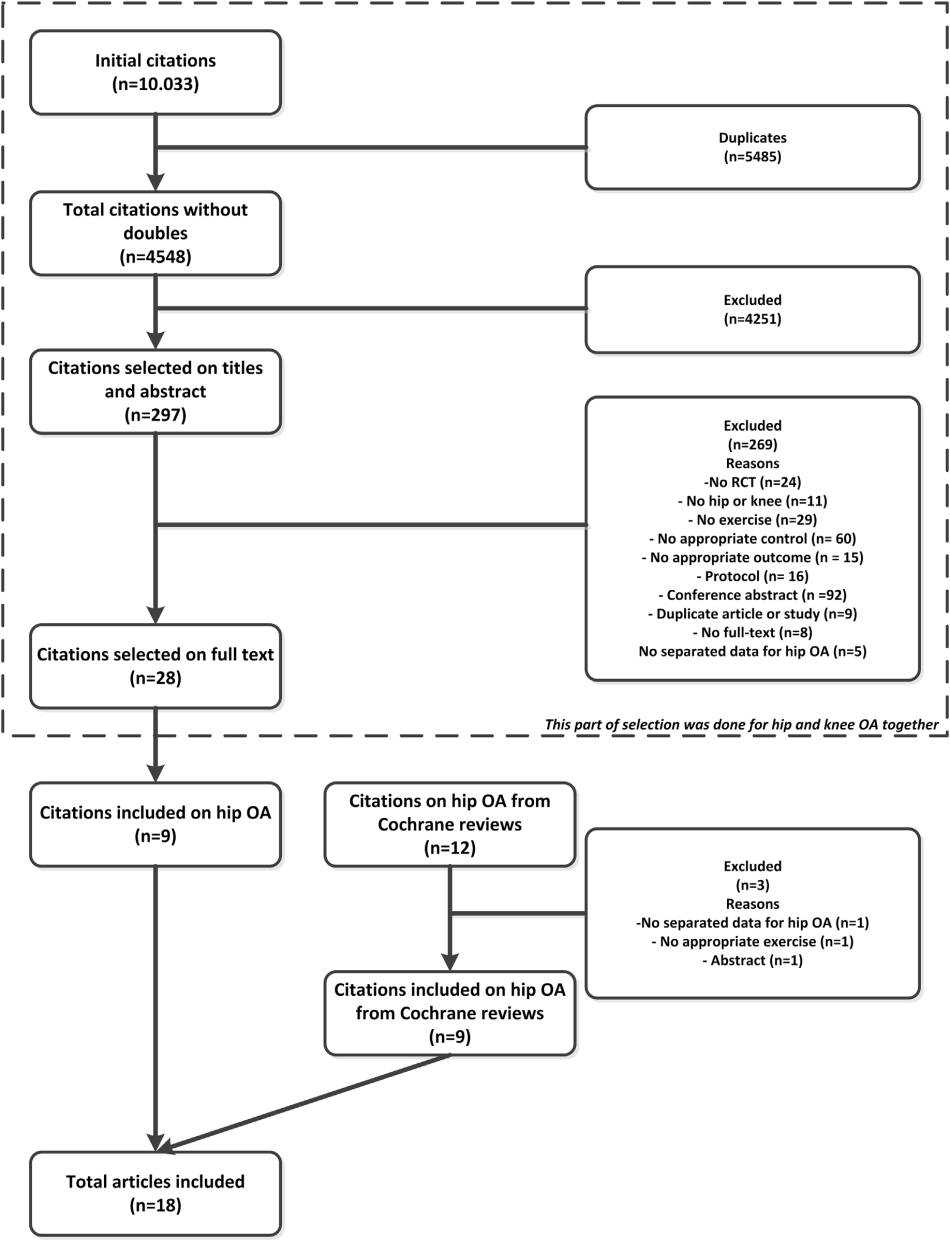
Flowchart.

The literature search and selection procedure were initially done for hip OA and knee OA together. For the present study, we only used the references of hip OA. In total 4548 references were found after the removal of duplicates. The titles and abstracts of these references were screened using the selection criteria and 297 references were selected for full-text reading. 28 references were included of which nine references included patients with hip OA (Fig. 1). Three studies from the 12 included studies in the Cochrane reviews were excluded for following reasons: no data available separately for hip OA patients, only an abstract was provided, exercise was not physical therapy but Tai Chi. In total nine studies of the Cochrane reviews could be included in our present study [[16], [17], [18], [19], [20], [21], [22], [23], [24]], together with the nine references of the literature search [[25], [26], [27], [28], [29], [30], [31], [32], [33]], so in total 18 studies were included.

### 3.2 Characteristics of included studies

Patient population. Number of patients per group ranged between 5 and 102 patients. In 7 studies, the smallest group included less than 25 patients. In most trials, patients were diagnosed using the ACR criteria for hip OA: clinical (n ​= ​8), radiological (n ​= ​3), clinical and radiological (n ​= ​3), or unclear (n ​= ​1) OA.

Interventions. All studies evaluated a land-based exercise, except for one study [22], which evaluated aquatic exercises. The duration of a treatment session varied from 30 to 120 ​min, frequency from 1 to 3 times a week and duration of the intervention from 5 to 16 weeks. Twelve studies were group-based, and six studies were individual based exercises. Control interventions were education (n ​= ​6), waiting list (n ​= ​5), usual care by general practitioner (n ​= ​6) and no intervention (n ​= ​1).

Outcomes. Pain was measured with the following instruments: Western Ontario and McMaster Universities Osteoarthritis Index (WOMAC, n ​= ​6), Hip disability and Osteoarthritis Outcome Score (HOOS, n ​= ​4), Visual Analogue Scale (VAS, n ​= ​4), Numeric Rating Scale (NRS, n ​= ​1), Brief Pain Inventory (BPI, n ​= ​1) and Impact of Rheumatic diseases on General Health and Lifestyle (IRGL, n ​= ​1). Function was measured with the following instruments: WOMAC (n ​= ​8), HOOS (n ​= ​4), IRGL (n ​= ​2), Disability Rating Index (DRI, n ​= ​1), Harris Hip Score (n ​= ​1), Health Assessment Questionnaire (HAQ, n ​= ​1) and 6-min walking test (n ​= ​1). More characteristics of the included studies can be found in Table 1.

**Table 1:** Characteristics of included studies.

Study	Population	Intervention	Control	Measurements^+^
V Baar 1998 [24]	Clinical ACR	Exercise (n ​= ​35). Individual physiotherapy program (12 weeks, 1–3x/week, 30-min sessions) ​+ ​GP education ​+ ​medication if necessary.	GP education ​+ ​medication if necessary (n ​= ​33).	***After treatment:*** Function: IRGL Pain: VAS pain past week
Hopman-Rock 2000 [20]	ACR	Exercise (n ​= ​11). Group sessions (6 weeks, 1x/week, 60-min classes) +1x/week education.	Waiting list (n ​= ​13).	***After treatment:*** Function: IRGL-mobility Pain: IRGL-pain
Foley 2003 [18]	Radiological ACR	Exercise (n ​= ​6). Group sessions (6 weeks, 2x/week 30-min classes)	Waiting list (n ​= ​12). Telephone call every 2 weeks.	***After treatment:*** Function: WOMAC Pain: WOMAC
Stener-Victorin 2004 [22]	Radiological ACR Patients on waiting list for hip replacement	Aquatic exercise (n ​= ​15). Group session (5 weeks, 2x/week, 30 ​min) ​+ ​education. Two group meetings lasting 2 ​h each concerning hip anatomy, disease process, and advice on physical activities.	Education (n ​= ​15). Two group meetings lasting 2 ​h each concerning hip anatomy, disease process, and advice on physical activities.	***At* 6 months *after treatment*** Function: Disability Rating Index Pain: VAS
Tak 2005 [23]	Clinical ACR	Exercise (n ​= ​55). Group session (8 weeks, 1x/week strengthening ​+ ​home program, 60-min) ​+ ​education.	GP care (n ​= ​54).	***After treatment:*** Function: Harris hip score Pain: VAS
Fernandes 2010 [17]	Radiological ACR and symptoms (Harris Hip Score 60–95)	Exercise (n ​= ​55). Individually based (12 weeks, 2x/week) ​+ ​patient education	Patient education (n ​= ​54).	***After treatment and at* 6 months *after treatment*** Function: WOMAC Pain: WOMAC
Juhakoski 2011 [21]	Radiological and clinical ACR, K-L grade >1	Exercise (n ​= ​60). Group sessions (12 weeks,1x/week, 45 ​min, +4 booster sessions 1 year later) ​+ ​GP-care	GP-care (n ​= ​60).	***After treatment and at* 9 months *after treatment:*** Function: WOMAC Pain: WOMAC
French 2013 [19]	Radiological and clinical ACR	Exercise (n ​= ​45). Individually provided ‘standardized’ exercise program (8 weeks, 6–8 sessions, 30-min) ​+ ​daily home exercise program (aerobic walking/cycling/swimming 30 ​min)	Waiting list (n ​= ​43).	***After treatment:*** Function: WOMAC-PF Pain: NRS
Abbott 2013 [16]	Clinical ACR	Exercise (n ​= ​22). Individually provided by physiotherapist, 50 ​min (9 weeks, 7 sessions +2 booster sessions week 16).	GP care (n ​= ​23).	***At* 8 months *after treatment:*** Function: WOMAC Pain: WOMAC
Villadsen 2014 [32]	Scheduled for hip replacement because of symptomatic OA	Exercise (n ​= ​43). Group sessions of neuromuscular training (8 weeks, 2x/week, 60 ​min) ​+ ​education (written information, also on various exercises)	Education (n ​= ​41). Written information, also on various exercises.	***After treatment:*** Function: HOOS Pain: HOOS
Kraus 2014 [28]	Clinical ACR	Exercise (n ​= ​71). Group sessions (12 weeks, 1x/week, 60–90 ​min, 2x/week home exercises, 30–40 ​min).	Control (n ​= ​69). No intervention.	***After treatment:*** Function: WOMAC Pain: WOMAC
Svege 2013/2015 [30]	Radiological and clinical ACR	Exercise (n ​= ​55). Individually provided by physiotherapist (12 weeks, 2–3x/week) ​+ ​education	Education (n ​= ​54).	***After treatment and at* 6 months *after treatment*** Function: WOMAC Pain: WOMAC
Teirlinck 2016 [31]	Clinical ACR	Exercise (n ​= ​101). Individual therapy (12 weeks, 12 sessions, 3 booster sessions in 5th, 7th and 9th month) ​+ ​GP care.	GP care (n ​= ​102).	***After treatment and* 6 months *after treatment:*** Function: HOOS∗ Pain: HOOS∗ ∗scores are reversed
Hermann 2016 [27]	Scheduled for hip replacement	Exercise (n ​= ​40). Group sessions of pre-operative progressive explosive resistance training (10 weeks, 2x/week, 60 ​min).	Usual care (n ​= ​40).	***After treatment:*** Function: HOOS Pain: HOOS
Saw 2016 [29]	Waiting list for hip replacement, radiological and clinical ACR	Exercise (n ​= ​14). Group sessions by physiotherapist (6 weeks, 1x/week, 120 ​min) ​+ ​education.	Usual care (n ​= ​16).	**After treatment and 6 months after treatment:** Function: Health Assessment Questionnaire - functional disability index Pain: Brief Pain Inventory
Bieler 2016 [25]	Clinical ACR, age> 60	Exercise (n ​= ​50). Group sessions, strengthening/resistance exercises (16 weeks, 3x/week, 60 ​min).	Counseling ​+ ​education (n ​= ​52).	***After treatment and* 8 months *after treatment*** Function:6MWT Pain: no data available
Chopp-Hurley 2017 [26]	Clinical ACR	Exercise (n ​= ​5). Group sessions (12 weeks, 3x/week, 60 ​min).	Waiting list (n ​= ​5).	**After treatment:** Function: HOOS Pain: HOOS
Thompson 2020 [33]	Radiological hip OA and pain and loss of function	Exercise (n ​= ​21). Groups sessions, strengthening/flexibility/endurance exercises (12 weeks, 3x per week, 60 ​min)	Waiting list (n ​= ​10).	**After treatment:** Function: WOMAC Pain: VAS

+ In this table we indicate the measurements and time of measurements that we used in the results. Most studies mentioned more outcomes or times of measurements. Abbreviations: GP ​= ​general practitioner, ACR ​= ​American College of Rheumatology, IRGL ​= ​invloed van Reuma op Gezondheid en Leefwijze (Influence of rheumatic diseases on Health and lifestyle), VAS ​= ​visual analogue scale, WOMAC= Western Ontario and McMaster Universities Osteoarthritis Index, PF=Physical Function subscale, NRS ​= ​numeric rating scale. HOOS= Hip disability and Osteoarthritis Outcome Score.

### 3.3 Risk of bias assessment

The risk of bias of each domain of each study can be found in Table 2. Overall, 13 studies scored a low of risk of bias, two studies a moderate risk of bias and three studies scored a high risk of bias. Due to the nature of the intervention, none of the studies was able to blind their participants, personnel, or outcome assessors (which were the participants for most outcomes). Therefore, all studies scored a high risk of bias on the two items of blinding, even if we had little or no information on these items. Only one study [25] reported that patients did not have a treatment preference and was therefore scored as low risk of bias on the blinding items.

**Table 2:** Risk of bias assessment.

Study	Random sequence generation	Allocation concealment	Blinding of participants and personnel	Blinding of outcome assessment	Incomplete outcome data	Selective reporting	Other bias	Overall risk of bias#
V Baar 1998 [24]	**+**	**+**	**-**	**-**	**+**	**?**	**+**	**Low**
Hopman-Rock 2000 [20]	**?**	**?**	**-**	**-**	**?**	**?**	**+**	**High**
Foley 2003 [18]	**+**	**+**	**-**	**-**	**+**	**?**	**?**	**Low**
Stener-Victorin 2004 [22]	**+**	**?**	**-**	**-**	**-**	**+**	**+**	**High**
Tak 2005 [23]	**+**	**?**	**-**	**-**	**+**	**?**	**+**	**High**
Fernandes 2010 [17]	**+**	**+**	**-**	**-**	**+**	**+**	**+**	**Low**
Juhakoski 2011 [21]	**+**	**+**	**-**	**-**	**+**	**?**	**+**	**Low**
French 2013 [19]	**+**	**+**	**-**	**-**	**+**	**+**	**+**	**Low**
Abbott 2013 [16]	**+**	**+**	**-**	**-**	**+**	**+**	**?**	**Low**
Villadsen 2014 [32]	**+**	**+**	**-**	**-**	**+**	**+**	**+**	**Low**
Kraus 2014 [28]	**+**	**+**	**-**	**-**	**+**	**+**	**+**	**Low**
Svege 2013/2015 [30]	**+**	**+**	**-**	**-**	**+**	**+**	**+**	**Low**
Teirlinck 2016 [31]	**+**	**+**	**-**	**-**	**+**	**+**	**+**	**Low**
Hermann 2016 [27]	**+**	**+**	**-**	**-**	**+**	**+**	**?**	**Low**
Saw 2016 [29]	**+**	**?**	**-**	**-**	**?**	**+**	**+**	**Moderate**
Bieler 2016 [25]	**+**	**+**	**+**	**+**	**+**	**+**	**+**	**Low**
Chopp-Hurley 2017 [26]	**+**	**+**	**-**	**-**	**?**	**+**	**+**	**Moderate**
Thompson 2021 [33]	**+**	**?**	**-**	**-**	**+**	**+**	**+**	**Low**

# Low RoB: randomization appropriate ​+ ​concealed ​+ ​ITT analysis; high RoB: <3 items low risk; moderate RoB: all else.

#### 3.3.1 Cumulative meta-analysis

A funnel plot was created using function post-treatment as outcome, because most studies reported this outcome (15 studies). The funnel plot did not show apparent evidence of publication bias, see figure A in supplement.

**Fig. 2:**
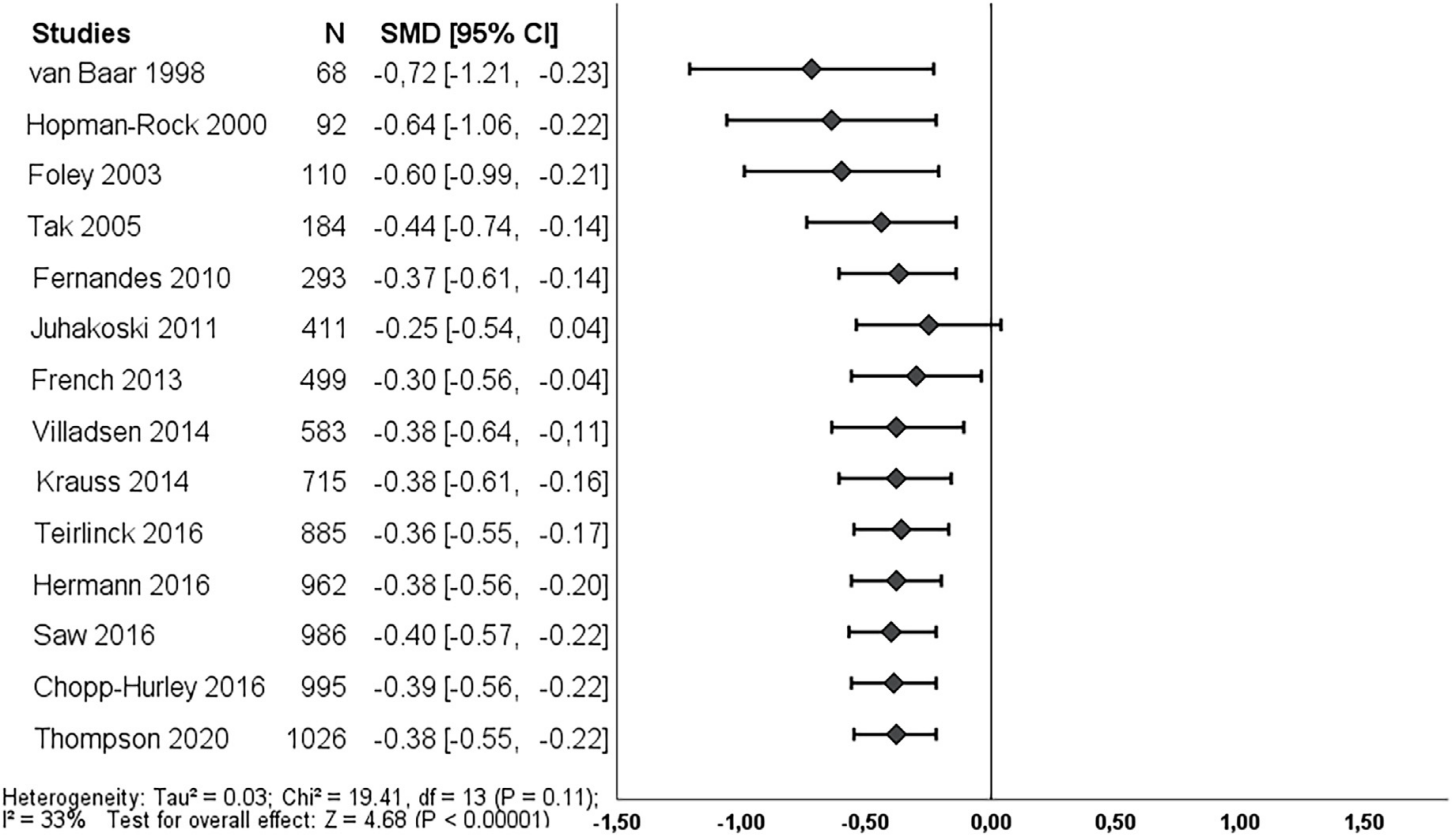
Cumulative meta-analysis on pain post-treatment, Footnote: each line represents the number of all participants and the pooled effect of the named study and studies of lines above (cumulative). For example, the line Foley 2003, shows the pooled effect (SMD and 95% CI) and number of participants (N) of the studies: van Baar 1998, Hopman-Rock 2000 and Foley 2003. The bottom line (Thompson 2020), is the pooled effect of all included studies.

Post-treatment, 14 studies reported on pain and 15 studies reported on function. We found a clinically worthwhile effect of exercise therapy on pain (SMD -0.38, 95% CI: 0.55 to −0.22) and this effect was already statistically significant in the first study in 1998 (Fig. 2). The effect could not be classified as *clearly* clinically worthwhile since the 95% CI did cross the threshold of SMD -0.37. Further studies showed that the direction of the effect estimate is consistent and only resulted in a smaller and more precise effect estimate in the cumulative meta-analysis. Overall, exercise therapy showed an unclear clinical worthwhile effect on function post-treatment (SMD -0.31, 95% CI -0.49 to −0.11), which became statistically significant in 2014 (Fig. 4).

**Fig. 3:**
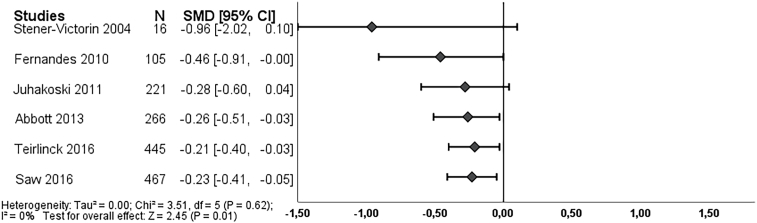
Cumulative meta-analysis on pain long-term, Footnote: each line represents the number of all participants and the pooled effect of the named study and studies of lines above (cumulative). For example, the line Juhakoski 2011, shows the pooled effect (SMD and 95% CI) and number of participants (N) of the studies: Stener-Victorin 2004, Fernandes 2010 and Juhakoski 2011. The bottom line (Saw 2016), is the pooled effect of all included studies.

Long-term outcome, six and seven studies respectively, reported on pain and function at 6–9 months after treatment. We found an overall effect on pain in favor of exercise therapy (SMD -0.23, 95% CI: 0.41 to −0.05) (Fig. 3), which became statistically significant in 2013. Exercise therapy showed an effect on function (SMD -0.29, 95% CI: 0.45 to −0.12), and this effect became statistically significant in 2010 (Fig. 5). Both effect estimates were regarded as unclear clinically worthwhile effects.

**Fig. 4:**
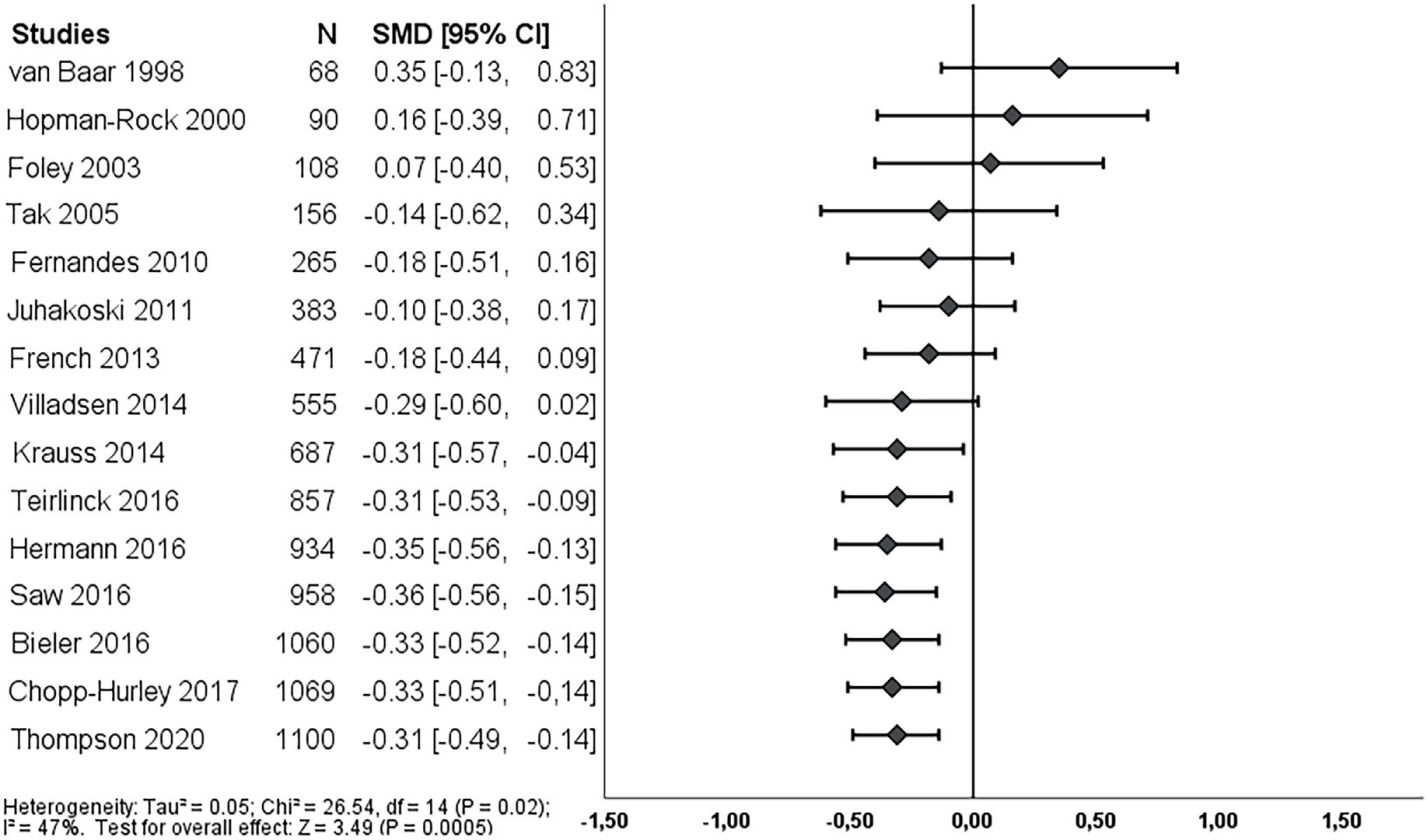
Cumulative meta-analysis on function post-treatment, Footnote: each line represents the number of all participants and the pooled effect of the named study and studies of lines above (cumulative). For example, the line Foley 2003, shows the pooled effect (SMD and 95% CI) and number of participants (N) of the studies: van Baar 1998, Hopman-Rock 2000 and Foley 2003. The bottom line (Thompson 2020), is the pooled effect of all included studies.

**Fig. 5:**
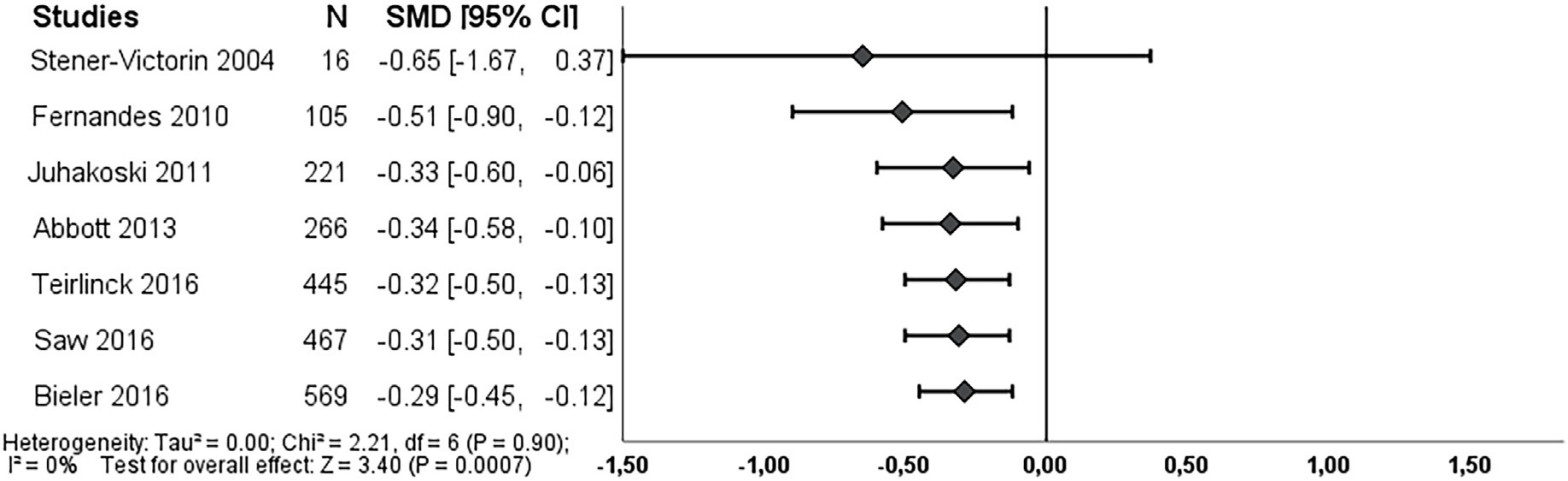
Cumulative meta-analysis on function long-term, Footnote: each line represents the number of all participants and the pooled effect of the named study and studies of lines above (cumulative). For example, the line Juhakoski 2011, shows the pooled effect (SMD and 95% CI) and number of participants (N) of the studies: Stener-Victorin 2004, Fernandes 2010 and Juhakoski 2011. The bottom line (Bieler 2016), is the pooled effect of all included studies.

The quality of evidence was moderate for function post treatment (downgrading for inconsistency) and high for pain post treatment, pain, and function at 6–9 months after treatment (no downgrading).

### 3.4 Extended funnel plots

**Fig. 6:**
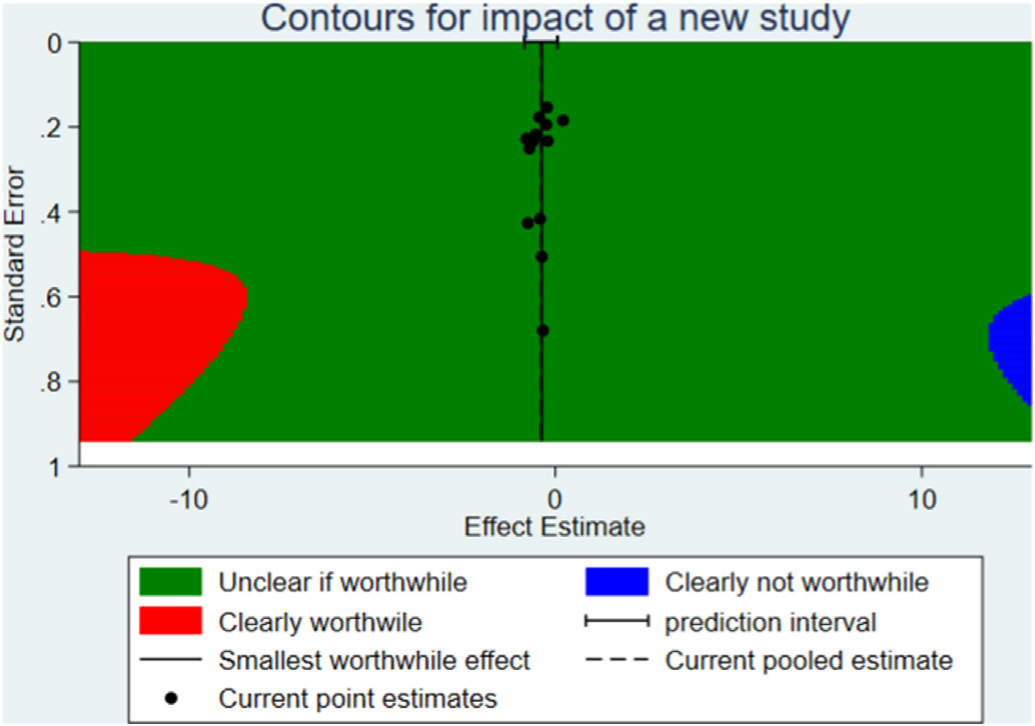
Extended funnel plot on pain post treatment.

We conducted extended funnel plots for pain and function post-treatment (Fig. 6 and figure B in supplement). These plots show that a new study need to have a large effect estimate to change the pooled effect estimate (and 95% CI) from an unclear (green region) to a clearly clinically worthwhile effect estimate (red region).

To estimate how large the sample size of a new study therefore needs to be, we simulated a cumulative meta-analysis by adding a fictional new trial using the current overall effect estimate (−0.38) of pain post-treatment. We calculated that the new trial should include 74,843 participants to change the overall effect estimate to a clearly clinically worthwhile effect.

## 4 Discussion

We found a clinically worthwhile effect on pain on short term and unclear clinically worthwhile effects on function on short term and pain and function on long term in our cumulative meta-analysis of exercise therapy compared to no or minimal treatment for patients with hip OA. Although these effects estimates were already statistically significant after the first study (pain short term) or after multiple studies, this effect is not yet a *clearly* clinically worthwhile effect. This is because the effect estimates and 95% CI of pain and function are respectively under but close, and above our predetermined value of a clinically worthwhile effect (SMD ​≤ ​−0.37). By simulating the effect of a new trial on current evidence, we concluded only an unrealistically large trial would result in an overall pooled effect estimate and confidence interval of clinically significance. This means, that the studies done so far show us that exercise therapy in hip OA patients has a modest effect on pain and function, possibly just clinically worthwhile. If we assume that this effect is the true effect, performing more studies to proof this to be clearly clinically worthwhile, will cost a lot of effort (and money) from researchers and patients while the value of this effort is questionable. Therefore, we would consider not to perform new trials on the effect of exercise therapy on hip osteoarthritis (compared to no or minimal treatment) but instead, focus on which type of exercises are most effective or which patients benefit most of exercise therapy. Earlier systematic reviews already concluded that there is little evidence on moderators [34] or type of exercises [35] on the effect of exercise therapy for hip OA. Recently though, some of the results of a large individual participant data meta-analysis on moderators of exercise therapy in knee and hip OA were presented and showed that patients with more pain or functional limitations at baseline respond slightly better to exercise therapy [36]. Unfortunately, this analysis was not done for hip OA patients alone. A recent study in a large cohort of patients with hip OA following a program with education and supervised exercise found that patients with better mental well-being and fewer comorbidities are more likely to be a responder (improvement of pain ≥30% from baseline) to this program [37]. Also, in females they found that attending the education lecture and more supervised exercise made it more likely to respond to the program and that females with symptoms at other joints were less likely to respond. Overall, there is not enough evidence to advice health care providers which hip OA patients benefit from which exercises, what duration, at what intensity and frequency.

### 4.1 Strength and limitations

Our results are consistent with earlier systematic reviews [6,8,38], but we are not aware of another cumulative meta-analysis on exercise therapy in patients with hip OA. Looking at the consistency within our research and our results, two studies were diverging. Firstly, one study did not found an effect of exercise therapy on function at short term [24]. A possible explanation could be that the included patients had an early phase of OA, since they had complaints for less than 1 year. Secondly, another study did not found a difference in WOMAC pain and function post-treatment [21]. At baseline the control group had a higher WOMAC pain score than the exercise group and, in addition, it seems that patients in the exercise group had more pain and worse function directly post-treatment than at baseline, while the control group had less pain and better function (although not statistically significant). These findings can possibly be explained by a low pain score at baseline (in both groups, but lower in the exercise group) since pain at baseline is a possible moderator of the effect of exercise [36].

Nonetheless, all other studies found an effect on both pain and function and quality of evidence was considered high (with exception of function short term). Furthermore, we followed the international recognized guidelines of Cochrane to perform this systematic review. By adding the cumulative meta-analysis, we tried to give more insight in the effort already done by researchers and the value of adding more research.

Other limitations of this review are the differences in intensity and duration of the exercises between all studies, which makes it more difficult to compare. In addition, in the included studies, blinding was not possible because of the intervention and patient reported outcomes. Thus, none of the studies blinded their participants and therefore all studies scored high risk at this item in the risk of bias assessment. Nevertheless, we decided that one study could be scored a low risk on the blinding items because patients were asked about their treatment preference and did not report a treatment preference. This decision is debatable, but since this study would also score an overall low risk bias without this item, it did not affect the cumulative meta-analysis.

## 5 Conclusion

Exercise therapy for patients with hip OA is effective, but the effect is small and not clearly clinically worthwhile. It is unrealistic that by performing more trials we can establish with certainty that the effect will become clearly worthwhile. We therefore recommend future trials to focus on which patients benefit most of exercise therapy and/or what kind of exercise therapy is most effective.

## Author contributions

Concept, AV, SB.; methodology, AV, SB, CT, MF.; formal analysis, CT, AV, ER, JR, MM, LR, SB, MF.; writing—original draft preparation, CT.; writing—review and editing, AV, ER, JR, MM, LR, SB, MF.; supervision, AV, SB; All authors have read and agreed to the submitted version of the manuscript.

## Funding

This research received no external funding.

## Data statement

All data is available on request.

## Declaration of competing interest

Dr. Bierma-Zeinstra reports grants from 10.13039/100018286Dutch Arthritis Society, the 10.13039/501100001826Netherlands Organisation for Health Research and Development, and 10.13039/100006939EU, personal fees from Osteoarthritis & Cartilage, from 10.13039/100004319Pfizer and Infirst health care, outside the submitted work. The other authors certify no conflict of interest in connection with the submitted article.
