# Supplementary material for: Effect of exercise therapy in patients with hip osteoarthritis: A systematic review and cumulative meta-analysis
Source: Osteoarthr Cartil Open. 2023 Jan 19;5(1):100338. doi: 10.1016/j.ocarto.2023.100338 (PMC9932106; doi:10.1016/j.ocarto.2023.100338)
Supplement: Multimedia component 1 [file mmc1.docx]

**Supplement**

Figure A. Funnel plot (on publication bias).


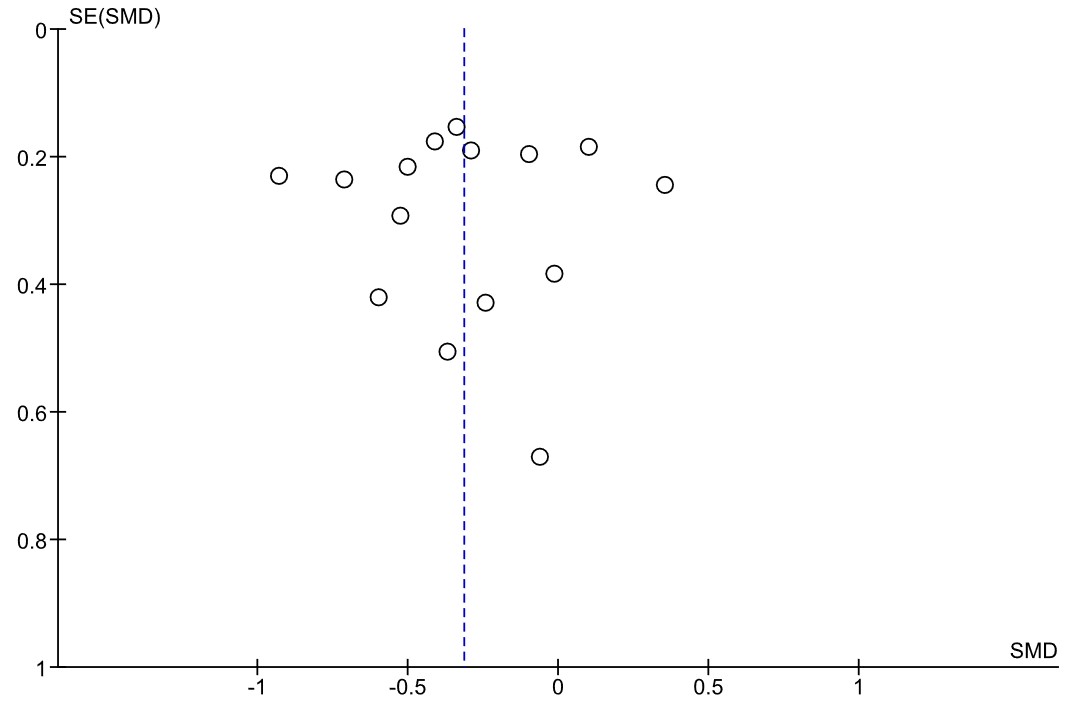


Figure B. Extended funnel plot on function post-treatment


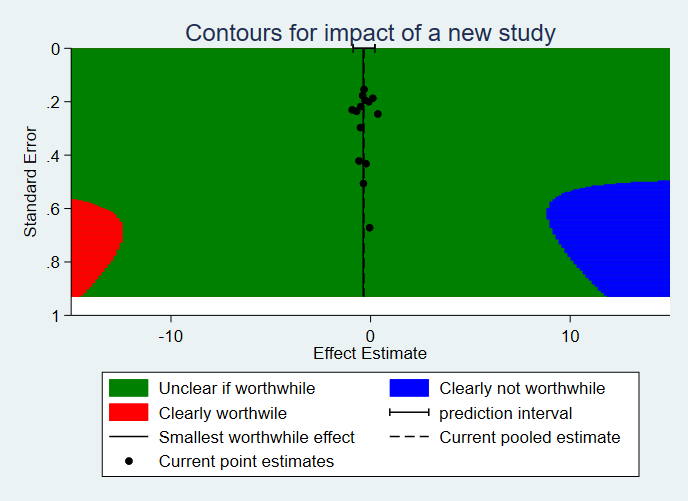


Syntax of literature search

**Embase.com**

(osteoarthritis/exp OR (osteoarthr* OR (degenerative NEAR/2 arthritis) OR arthrosis):ab,ti,kw) AND (Hip/exp OR 'hip osteoarthritis'/de OR (hip*):ab,ti,kw) AND (exercise/exp OR fitness/de OR 'exercise test'/de OR 'exercise tolerance'/de OR Sport/exp OR pliability/de OR 'physical activity, capacity and performance'/exp OR physiotherapy/exp OR rehabilitation/exp OR (exertion* OR exercis* OR sport* OR ((physical or motion) NEAR/5 (fitness or therap*)) OR (physical* NEAR/2 endur*) OR ((strength* or isometric* or isotonic* or isokinetic* or aerobic* or endurance or weight*) NEAR/5 (train*)) OR aquatic* OR exercis* OR physiotherap* OR manipulat* OR kinesiotherap* OR rehab* OR (skate* or skating) OR running OR jogging OR treadmill* OR swim* OR bicycl* OR (cycle* or cycling) OR walk* OR (row or rows or rowing) OR Muscle-strength* OR Balneotherap* OR hydrotherap* OR pool-therap*):ab,ti,kw) AND ('crossover procedure'/exp OR 'double blind procedure'/exp OR 'randomized controlled trial'/exp OR 'single blind procedure'/exp OR (random* OR factorial* OR (crossover* or cross-over*) OR placebo* OR (doubl* NEXT/1 blind*) OR (singl* NEXT/1 blind*) OR assign* OR allocat* OR volunteer* ):ab,ti) NOT ([animals]/lim NOT [humans]/lim) AND [2013-2030]/py

**Medline Ovid**

(exp osteoarthritis/ OR (osteoarthr* OR (degenerative ADJ2 arthritis) OR arthrosis).ab,ti,kf.) AND (Hip/ OR "osteoarthritis, hip"/ OR (hip*).ab,ti,kf.) AND (exp Physical Exertion/ OR exp Physical Fitness/ OR exp Exercise Test/ OR exp Exercise Tolerance/ OR exp Sports/ OR exp pliability/ OR exp Physical Endurance/ OR (exertion* OR exercis* OR sport* OR ((physical or motion) ADJ5 (fitness or therap*)) OR (physical* ADJ2 endur*) OR ((strength* or isometric* or isotonic* or isokinetic* or aerobic* or endurance or weight*) ADJ5 (train*)) OR aquatic* OR exercis* OR physiotherap* OR manipulat* OR kinesiotherap* OR rehab* OR (skate* or skating) OR running OR jogging OR treadmill* OR swim* OR bicycl* OR (cycle* or cycling) OR walk* OR (row or rows or rowing) OR Muscle-strength* OR Balneotherap* OR hydrotherap* OR pool-therap*).ab,ti,kf.) AND (randomized controlled trial.pt. OR controlled clinical trial.pt. OR randomized.ab. OR placebo.ab. OR drug therapy.fs. OR randomly.ab. OR trial.ab. OR groups.ab. ) NOT (exp animals/ NOT humans/) AND 2013:2030.(sa_year).

**Cochrane (filter: from 2013 onwards)**

((osteoarthr* OR (degenerative NEAR/2 arthritis) OR arthrosis):ab,ti) AND ((hip*):ab,ti) AND ((exertion* OR exercis* OR sport* OR ((physical OR motion) NEAR/5 (fitness OR therap*)) OR (physical* NEAR/2 endur*) OR ((strength* OR isometric* OR isotonic* OR isokinetic* OR aerobic* OR endurance OR weight*) NEAR/5 (train*)) OR aquatic* OR exercis* OR physiotherap* OR manipulat* OR kinesiotherap* OR rehab* OR (skate* OR skating) OR running OR jogging OR treadmill* OR swim* OR bicycl* OR (cycle* OR cycling) OR walk* OR (row OR rows OR rowing) OR Muscle-strength* OR Balneotherap* OR hydrotherap* OR pool-therap*):ab,ti)

**Web of science**

TS=(((osteoarthr* OR (degenerative NEAR/1 arthritis) OR arthrosis)) AND ((hip*)) AND ((exertion* OR exercis* OR sport* OR ((physical or motion) NEAR/4 (fitness or therap*)) OR (physical* NEAR/1 endur*) OR ((strength* or isometric* or isotonic* or isokinetic* or aerobic* or endurance or weight*) NEAR/4 (train*)) OR aquatic* OR exercis* OR physiotherap* OR manipulat* OR kinesiotherap* OR rehab* OR (skate* or skating) OR running OR jogging OR treadmill* OR swim* OR bicycl* OR (cycle* or cycling) OR walk* OR (row or rows or rowing) OR Muscle-strength* OR Balneotherap* OR hydrotherap* OR pool-therap*)) AND ((random* OR factorial* OR (crossover* or cross-over*) OR placebo* OR (doubl* NEAR/1 blind*) OR (singl* NEAR/1 blind*) OR assign* OR allocat* OR volunteer* )) ) AND py=(2013-2030)

**CINAHL EBSCOhost**

(MH osteoarthritis+ OR TI (osteoarthr* OR (degenerative N1 arthritis) OR arthrosis) OR AB (osteoarthr* OR (degenerative N1 arthritis) OR arthrosis)) AND (MH Hip OR MH "osteoarthritis, hip" OR (hip*)) AND (MH exertion+ OR MH Physical Fitness+ OR MH Exercise Test+ OR MH Exercise Tolerance+ OR MH Sports+ OR MH pliability+ OR MH Physical Endurance+ OR TI (exertion* OR exercis* OR sport* OR ((physical or motion) N4 (fitness or therap*)) OR (physical* N1 endur*) OR ((strength* or isometric* or isotonic* or isokinetic* or aerobic* or endurance or weight*) N4 (train*)) OR aquatic* OR exercis* OR physiotherap* OR manipulat* OR kinesiotherap* OR rehab* OR (skate* or skating) OR running OR jogging OR treadmill* OR swim* OR bicycl* OR (cycle* or cycling) OR walk* OR (row or rows or rowing) OR Muscle-strength* OR Balneotherap* OR hydrotherap* OR pool-therap*) OR AB (exertion* OR exercis* OR sport* OR ((physical or motion) N4 (fitness or therap*)) OR (physical* N1 endur*) OR ((strength* or isometric* or isotonic* or isokinetic* or aerobic* or endurance or weight*) N4 (train*)) OR aquatic* OR exercis* OR physiotherap* OR manipulat* OR kinesiotherap* OR rehab* OR (skate* or skating) OR running OR jogging OR treadmill* OR swim* OR bicycl* OR (cycle* or cycling) OR walk* OR (row or rows or rowing) OR Muscle-strength* OR Balneotherap* OR hydrotherap* OR pool-therap*)) AND (PT randomized controlled trial OR PT controlled clinical trial OR AB (randomized OR placebo OR randomly OR trial OR groups )) NOT (MH animals NOT MH humans) AND PY 2013-2030
